# Effectiveness and priority of irradiation and six NSAIDs in prevention heterotopic ossification after total hip arthroplasty: a network meta-analysis of randomized controlled studies

**DOI:** 10.3389/fphar.2025.1601349

**Published:** 2025-05-21

**Authors:** Fei Yang, Lei Wen, Changshun Chen, Qiao Zhao, Zhiwei Feng, Bin Ran, Xuwei Luo, Dongqin Xiao, Qifan Fang

**Affiliations:** ^1^ Nanchong Central Hospital, The Second Clinical Medical College of North Sichuan Medical College, Nanchong, China; ^2^ The Second Clinical Medical School, Lanzhou University, Lanzhou, China; ^3^ Department of Orthopedics and Trauma Surgery, Affiliated Hospital of Yunnan University, Kunming, China

**Keywords:** heterotopic ossification, total hip arthroplasty, prevention, network meta-analysis, NSAIDs

## Abstract

**Background:**

Heterotopic ossification (HO) involves the ectopic deposition of bone in soft tissues, frequently occurring as a complication post-hip trauma or surgery. To prevent HO following total hip arthroplasty (THA), irradiation has been extensively employed, alongside the use of nonsteroidal anti-inflammatory drugs (NSAIDs). Given the extensive range of NSAIDs available, determining the most effective NSAID or irradiation protocol for prophylaxis continues to be a matter of debate.

**Methods:**

Adhering to the PRISMA guidelines, a comprehensive search was conducted across PubMed, Embase, Cochrane Library, and Web of Science to identify relevant randomized controlled trials. To minimize bias in literature evaluation, two authors independently searched and assessed the articles. In cases of disagreement, a third author was consulted. We strictly implement the inclusion and exclusion criteria. Using the criteria for assessing bias in the Cochrane Collaboration Network, two writers independently evaluated the quality of the included studies. We systematically extracted and assessed data according to the level of evidence presented in the articles. A Bayesian network meta-analysis (NMA) was implemented to evaluate and contrast the efficacy of irradiation and six distinct NSAIDs in preventing HO after THA. The results were computed using the GEMTC package in R (V.4.4.1). The consistency of the model was tested using nodal analysis. The priority of drug efficacy was comprehensively evaluated using rank probability and the surface under the cumulative ranking curve (SUCRA). Stata 16 was used to assess publication bias, and sensitivity analysis was performed using the one-by-one elimination method. The protocol for this study was officially registered with the International Platform of Registered Systematic Review and Meta-analysis Protocols (INPLASY).

**Results:**

A total of 461 studies were identified, and 17 studies were finally included in the analysis. The meta-analysis incorporated data from 3,014 patients: 629 administered ibuprofen, 54 with naproxen, 117 receiving celecoxib, 426 on indomethacin, 295 treated with diclofenac, 45 on etoricoxib, 522 subjected to irradiation, and 926 serving as controls. These trials reported an average age ranging from 59 to 75 years, with males comprising 31.2%–63% of subjects. The total incidence rate of HO in all control groups was 55.2%. In terms of effectiveness, compared with the control, four strategies showed a low incidence of HO, including naproxen (OR = 0.08, 95% CrI 0.01–0.60), indomethacin (OR = 0.13, 95% CrI 0.04–0.41), diclofenac (OR = 0.06, 95% CrI 0.01–0.29), and irradiation (OR = 0.08, 95% CrI 0.02–0.3). Diclofenac was more beneficial than ibuprofen (OR = 0.10, 95% CrI 0.01–0.97). The probabilities derived from the surface under the cumulative ranking curve (SUCRA) algorithm are as follows: Diclofenac (78.0%), etoricoxib (71.6%), irradiation (67.3%), naproxen (66.7%), indomethacin (53.2%), celecoxib (38.8%), ibuprofen (18.6%), and a control group (6.8%). Because stronger evidence supports the efficacy of diclofenac. The most likely ranking for the effectiveness of preventing HO after THA is as follows: Diclofenac > etoricoxib > irradiation > naproxen > indomethacin > celecoxib > ibuprofen.

**Conclusion:**

In terms of preventive efficacy, diclofenac and etoricoxib demonstrated the most favorable performance in preventing HO after THA within this network meta-analysis. Irradiation, naproxen, and indomethacin are also satisfactory options, while ibuprofen is ineffective. Given the advantages shown by etoricoxib and celecoxib, further randomized controlled trials are recommended to clarify their effects. Our conclusions require confirmation through additional high-quality studies.

## Introduction

Heterotopic ossification is a pathological process of abnormal bone formation in extraosseous soft tissues (Balboni et al., 2006). Although its pathogenesis is not fully understood, most scientists agree that it is a reactivation of the bone formation process (Lachiewicz et al., 2024). The process is initiated by an inflammatory response and local progenitor cells, such as chondrocytes and osteoblasts, proliferate and differentiate in response to the inflammatory response and are ultimately remodeled and mature to form ectopic bone tissue. The imbalance of inflammatory factors and the initiation of the inflammatory response play an important role in the process of heterotopic ossification (Hwang et al., 2022; Wang et al., 2022). As a common complication after hip surgery, HO has an incidence of 15%–90% after primary total hip arthroplasty (Herzberg et al., 2024; Łęgosz et al., 2019). Especially in patients with femoral neck fracture, ankylosing spondylitis, previous hip fracture and hypertrophic osteoarthritis (Peng et al., 2020; Neitzke et al., 2024). It is also worthy of note that the incidence of HO following hip arthroscopy is 8.3% (Bedi et al., 2012). The bony structure formed by HO may compress peripheral nerves and blood vessels, resulting in limited joint motion, pain, and delayed functional recovery, which significantly increases the difficulty of postoperative rehabilitation and the economic burden, and severely weakens the efficacy of surgery and patient satisfaction (Rama et al., 2009; Wang et al., 2023a).

At present, clinical strategies employed for the prevention of HO principally comprise pre-/postoperative irradiation and pharmacological interventions (Puga et al., 2025). Irradiation has been demonstrated to reduce the risk of HO by inhibiting the osteogenic differentiation ability of progenitor cells (Heyd et al., 1995; Marziano et al., 1996), Cai et al. found that the overall incidence of HO after THA was low in patients treated with irradiation, and that irradiation was the most effective method to prevent HO after THA (Cai et al., 2019). Despite the broad consensus regarding the efficacy of irradiation in preventing HO following THA, considerable controversy persists with regard to the optimal radiation dose, timing, and potential carcinogenic risk (Heyd et al., 1997; van Leeuwen et al., 1998). NSAIDs have been shown to exert their anti-inflammatory effects by inhibiting prostaglandin synthesis. Indomethacin has long been regarded as the “gold standard” for preventing HO, and diclofenac has been shown to have a unique advantage in the reduction of severe HO (Brooker grade III-IV) (Macfarlane et al., 2008). In recent years, selective cyclooxygenase-2 inhibitors (e.g., celecoxib) have gained prominence in clinical use due to their improved gastrointestinal safety and long-lasting anti-inflammatory efficacy (Barbato et al., 2012; Lavernia et al., 2014; Oberberg et al., 2021). Migliorini et al. found that celecoxib was the most effective in reducing the incidence of HO, followed by diclofenac and indomethacin by traditional mata analysis (Migliorini et al., 2021). However, Shapira et al. also found by meta-analysis that non-selective and selective NSAIDs had comparable efficacy in the prevention of HO, yet were more effective than irradiation (Shapira et al., 2021). However, the majority of extant studies have focused on a single drug class or included only a limited number of interventions, and have not systematically quantified the difference in efficacy between different NSAIDs.

Notwithstanding the body of evidence that supports the preventive value of non-steroidal anti-inflammatory drugs (NSAIDs) in comparison with radiotherapy, a number of key controversies remain unresolved (Anaspure et al., 2025). Firstly, the boundary between the efficacy of traditional non-selective NSAIDs (e.g., ibuprofen, naproxen) and newer selective inhibitors (e.g., The following aspects require further clarification: firstly, the results obtained from different studies have been inconsistent, or even opposite; secondly, there is a lack of multifactor corrected analysis of the relative comparative advantage of irradiation versus pharmacological interventions. To this end, this study is the first to undertake a simultaneous integration of randomised controlled trial data from 3,014 postoperative THA patients based on the Bayesian network meta-analysis (NMA) framework to systematically compare the preventive efficacy of ibuprofen, naproxen, celecoxib, indomethacin, diclofenac, erliximab and radiotherapy. The optimal intervention programme was elucidated by probability ranking and multi-node evidence network construction. The objective of this study is 3-fold: firstly, to overcome the limitations of conventional two-by-two comparisons; secondly, to furnish orthopaedic surgeons with a foundation for selecting individualised preventive regimens based on a high level of evidence; and thirdly, to provide theoretical support for the direction of future clinical trials.

## Materials and methods

### Data sources and search strategy

In line with PRISMA recommendations, a meta-analysis of RCTs was performed using the PICO framework. Four databases—Web of Science, Embase, Cochrane Library, and PubMed—were searched using both MeSH terms and their free-form equivalents. For instance, the following search parameters were used to find naproxen: (Ossification, Heterotopic) AND (Arthroplasty, Replacement, Hip) AND (Randomized Controlled Trials as Topic) AND (Naproxen). The search was first carried out in September 2024 and then again on November 10th to make sure that any more research were included. We widened the search window to include all databases from their inception up until the current day. Here is the approach of searching the PubMed database: ((((((((((“Ossification, Heterotopic” [Mesh]) OR (Ossification, Ectopic)) OR (Ossification, Pathologic)) OR (Ossification, Pathological)) OR (Pathological Ossification)) OR (Pathologic Ossification)) OR (Heterotopic Ossification)) OR (Ectopic Ossification)) AND ((((((((((((((((((((“Arthroplasty, Replacement, Hip” [Mesh]) OR (Hip Replacement Arthroplasty)) OR (Replacement Arthroplasties, Hip)) OR (Replacement Arthroplasty, Hip)) OR (Hip Prosthesis Implantation)) OR (Hip Prosthesis Implantations)) OR (Implantation, Hip Prosthesis)) OR (Prosthesis Implantation, Hip)) OR (Arthroplasties, Hip Replacement)) OR (Arthroplasties, Replacement, Hip)) OR (Hip Replacement Arthroplasties)) OR (Arthroplasty, Hip Replacement)) OR (Hip Replacement, Total)) OR (Total Hip Arthroplasty)) OR (Arthroplasty, Total Hip)) OR (Hip Arthroplasty, Total)) OR (Total Hip Arthroplasties)) OR (Replacement, Total Hip)) OR (Total Hip Replacements)) OR (Total Hip Replacement))) AND ((((“Randomized Controlled Trials as Topic” [Mesh]) OR (Clinical Trials, Randomized)) OR (Trials, Randomized Clinical)) OR (Controlled Clinical Trials, Randomized))) AND ((((((((((((“Naproxen” [Mesh]) OR (Methoxypropiocin)) OR (Anaprox)) OR (Synflex)) OR (Proxen)) OR (Aleve)) OR (Naproxen Sodium)) OR (Sodium, Naproxen)) OR (Sodium Naproxenate)) OR (Naproxenate, Sodium)) OR (Naprosin)) OR (Naprosyn)).

To minimize bias in literature evaluation, two authors (FY and LW) independently searched and assessed the articles. In cases of disagreement, a third author (CC) was consulted. Our study adhered to the PICO model: P (patient) – adult patients scheduled for THA, I (intervention) – prevention of HO, C (comparison) – patients receiving ibuprofen, naproxen, celecoxib, indomethacin, diclofenac, etoricoxib, or irradiation therapy, and the control group, O (outcome) – primary outcome: incidence of HO; secondary outcome: gastrointestinal side effects.

### Inclusion and exclusion criteria

Studies were included if they involved patients undergoing THA and receiving ibuprofen, naproxen, celecoxib, indomethacin, diclofenac, etoricoxib, irradiation, control or placebo, compared with at least two of the eight regimens. Research with human participants that was published in randomized controlled trials prior to March 2025 and had a radiographic follow-up time of at least 12 weeks was only taken into consideration. Retrospective studies, case reports, conference proceedings, non-randomized cohort studies, and systematic reviews were all deemed ineligible.

### Quality assessment and data extraction

Using the criteria for assessing bias in the Cochrane Collaboration Network, two writers (FY and LW) evaluated the quality of the included studies. When there was a dispute, author CC. made the ultimate call. Two reviewers checked each other’s work after data extraction was complete. Author information, publication year, patient count, average age, male proportion, patient attrition rate, specifics of diagnosis and surgery, time and manner of administration, and occurrence of HO and gastrointestinal side effects were among the data extracted. This meta-analysis was registered with the INPLASY under registration number INPLASY2024100111.

Using a Bayesian framework and a random-effects model, we conducted a multi-treatment NMA (Lumley, 2002). In order to assess the frequency of HO, Brooker III and IV HO, and gastrointestinal side effects, we computed pooled estimates of odds ratios (OR) and 95% credible intervals (CrIs). The results were computed using the GEMTC package in R (V.4.4.1). The consistency of the model was tested using nodal analysis. Consistency was good when the *P*-value was more than 0.05. We ranked the eight strategies (irradiation, celecoxib, naproxen, diclofenac, etoricoxib, indomethacin, ibuprofen, and control) based on rank probabilities. The priority of drug efficacy was comprehensively evaluated using rank probability and the surface under the cumulative ranking curve (SUCRA). A heterogeneity test was also performed on the comparison results. An *I* ^2^ greater than 50% indicated significant heterogeneity. To evaluate the stability of the meta-analysis, Stata 16 was used to assess publication bias, a funnel plot was generated, and sensitivity analysis was performed using the one-by-one elimination method.

## Results

### Data retrieval and inclusion

**FIGURE 1:**
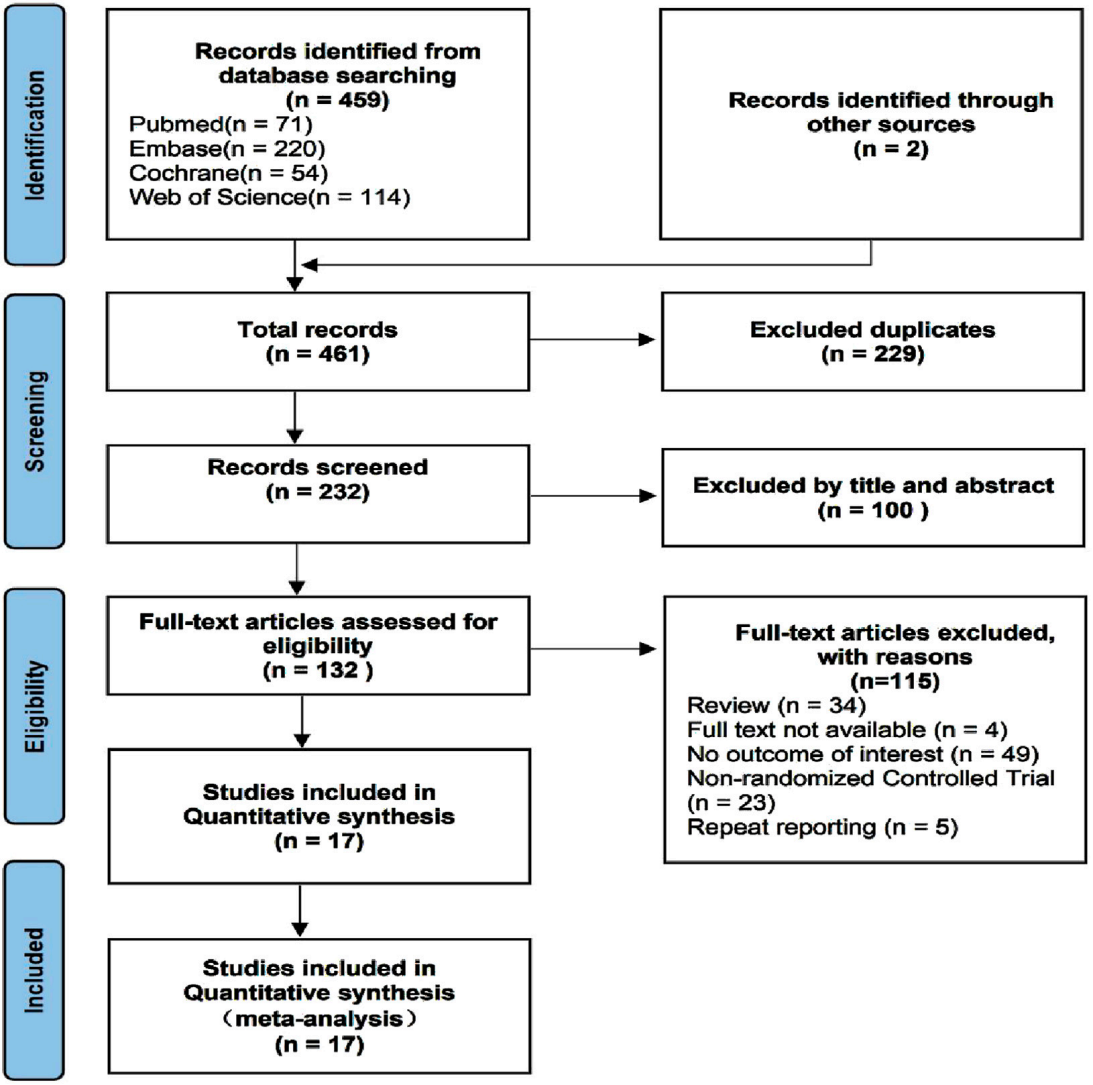
PRISMA flow diagram of screening process.

**FIGURE 2:**
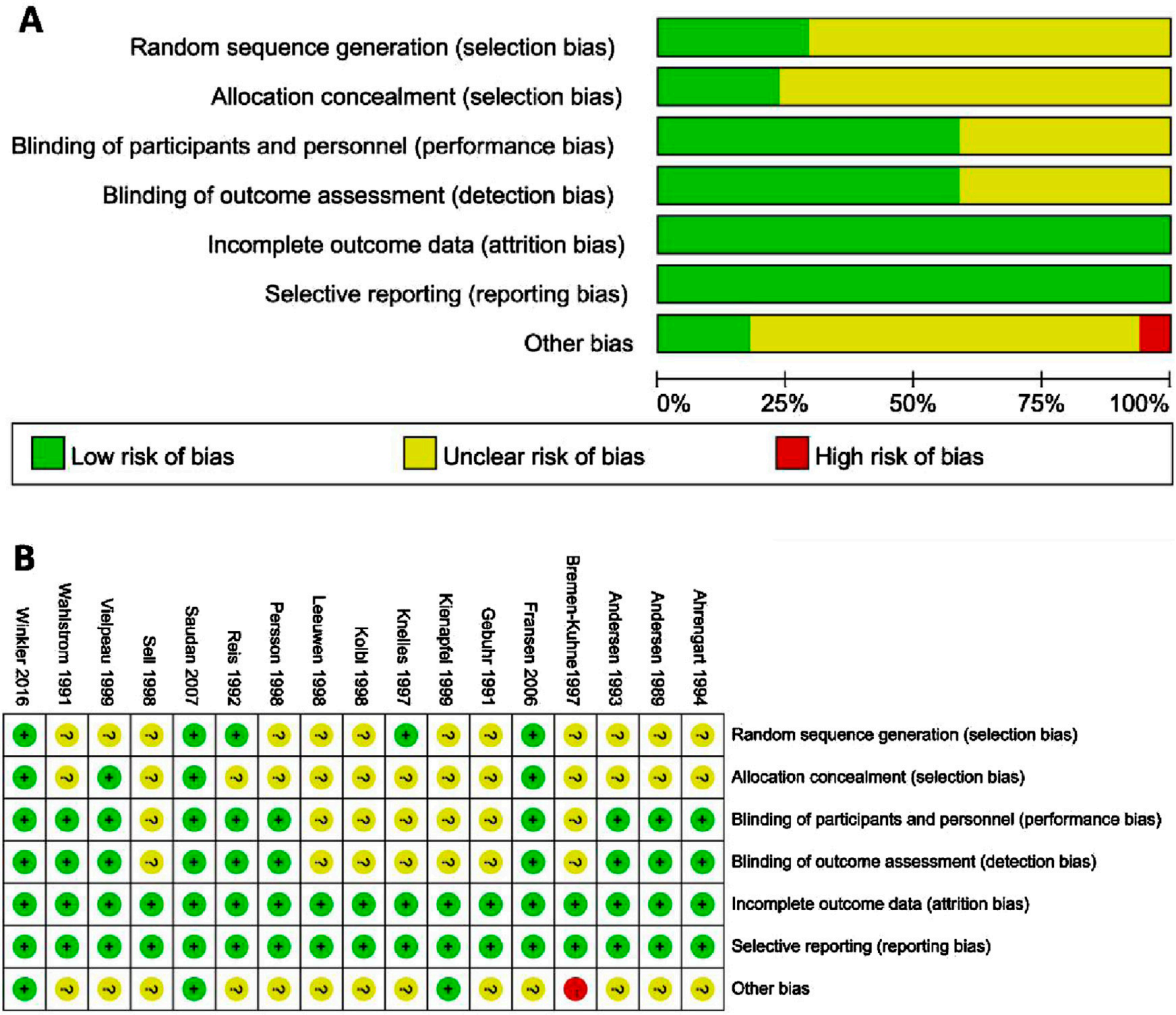
**(A)** Risk of bias. **(B)** Summary of risk of bias.

A total of 461 studies were identified, from which 229 duplicate articles were excluded. One hundred studies were screened by reviewing titles and abstracts. After excluding four articles for which full texts were unavailable, 132 articles were assessed in full. Of these, 34 reviews or meta-analyses, 49 articles lacking relevant information, and 23 non-randomized trials were excluded. The populations of five reports overlapped with other studies. Ultimately, 17 studies (van Leeuwen et al., 1998; Fransen et al., 2006; Persson et al., 1998; Ahrengart et al., 1994; Saudan et al., 2007; Vielpeau et al., 1999; Gebuhr et al., 1991; Reis et al., 1992; Wahlström et al., 1991; Sell et al., 1998; Winkler et al., 2016; Kjaersgaard-Andersen et al., 1993; Kjaersgaard-Andersen et al., 1989; Knelles et al., 1997; Kienapfel et al., 1999; Bremen-Kuhne et al., 1997; K̈olbl et al., 1998) were included in the analysis. These trials reported an average age ranging from 59 to 75 years, with males comprising 31.2%–63% of subjects, and a majority diagnosed with arthrosis. Baseline data are presented in Table 1, and clinical interventions and outcomes are presented in Table 2. The meta-analysis employed a total of 3,014 patients, with 629 assigned to the ibuprofen group, 54 to naproxen, 117 to celecoxib, 426 to indomethacin, 295 to diclofenac, 45 to etoricoxib, 522 to irradiation, and 926 to the control group. The incidence of HO in individuals who received or did not receive indole treatment was directly compared in these investigations. The PRISMA flowchart utilized by the researchers is shown in Figure 1, and the summary and risk of bias are depicted in Figure 2.

**TABLE 1:** Characteristics of the included studies.

Author/Ref	Period of inclusion	Region	Design	Patients N°		Male sex %		Age mean (SD)		Last radiological follow-up months		Patient lost to follow up N°	
				Intervention	Control or placebo	Intervention	Control or placebo	Intervention	Control or placebo	Intervention	Control or placebo	Intervention	Control or placebo
Fransen et al. (2006)	2002–2004	Australia and New Zealand	RCT	Ibuprofen: 452	Placebo: 450	54^a^	54^a^	66 (12)^a^	67 (11)^a^	6–12	6–12	27	22
Persson et al. (1998)	—	Sweden	RCT	G1 (Ibuprofen): 48 G2 (Ibuprofen): 48	Placebo: 48	50	50	—	—	12	12	G1: 2 G2: 0	3
Ahrengart et al. (1994)	1988–1989	Sweden	RCT	Ibuprofen: 30	Placebo:27	47.4 (All groups)		70 (7) (All averaged)		12	12	0	0
Saudan et al. (2007)	2001–2002	Switzerland	RCT	G1 (Celecoxib): 123 G2 (Ibuprofen): 127	None	G1:47 G2: 46	None	G1: 69 G2: 70	None	G1: 3 G2: 3	None	G1: 4 G2: 1	None
Vielpeau et al. (1999)	—	France	RCT	G1 (Naproxen): 28 G2 (Indomethacin): 28	Placebo: 28	—	—	G1: 66 (7.8) G2: 63.9 (8.7)	62.8 (9.6)	6	6	G1: 2 G2: 0	0
Gebuhr et al. (1991)	1987–1989	Denmark	RCT	Naproxen: 28	Placebo: 27	39	44	75	70	12	12	0	0
Reis et al. (1992)	1987	Germany	RCT	Diclofenac: 89	Placebo: 69	43 (All groups)		59 (All averaged)		24	24	—	—
Wahlstrom et al. (1991)	1985–1986	Sweden	RCT	Diclofenac: 50	Placebo: 48	60	60.8	71 (6.2)	70 (6.1)	3	3	1 (All groups)	
Sell et al. (1998)	1992–1993	Germany	RCT	G1 (Irradiation): 76 G2 (Diclofenac): 77	None	G1: 63 G2: 53	None	G1: 60.4 G2: 61.1	None	G1: 6 G2: 6	None	G1: 0 G2: 0	None
Winkler et al. (2016)	2011–2014	Germany	RCT	G1 (Etoricoxib): 47 G2 (Diclofenac): 48	None	G1: 53.2 G2: 54.2	None	G1: 60.2 G2: 61.9	None	G1: 6 G2: 6	None	G1: 2 G2: 4	
Kjaersgaard-Andersen et al. (1993)	1990–1991	Denmark	RCT	19	22	32	36	72	70	3	3	—	—
Kjaersgaard-Andersen et al. (1989)	1984–1986	Denmark	RCT	90	86	52 (All groups)		68 (All averaged)		12	12	—	—
Knelles et al. (1997) ^b^	1988–1994	Germany	RCT	G2^b^ (Indomethacin): 90 (Exited 4 from 94) G3 (Indomethacin): 113 (Exited 5 from 118 G4 (Irradiation): 101 (Exited 1 from 102) G5 (Irradiation): 95 G6 (Irradiation): 93	Control: 100	G2: 32.2 G3: 36.3 G4: 44.6 G5: 43.2 G6: 31.2	31	G2: 67 G3: 64.7 G4: 66.9 G5: 67.3 G6: 66.2	65.3	12	12	0	0
Kienapfel et al. (1999)	1992–1993	Germany	RCT	G1 (Irradiation): 49 G1 (Indomethacin): 55	Control: 50	G1: 46.9 G2: 40	24	G1: 63.9 G2: 64.4	66	18	18	G1: 0 G2: 0	0
Bremen-Kuhne et al. (1997)	1992–1994	Germany	RCT	G1 (Irradiation): 33 G2 (Indomethacin): 31	None	G1: 52.6 G2: 51.6	None	≥40 (50–59 is the majority)	None	12	None	0	None
van Leeuwen et al. (1998)	1989–1992	Netherlands	RCT	Irradiation: 41	Control: 16	31.3	29.3	66 (All averaged)		31	31	0	0
Kolbl et al. (1998)	1995–1996	Germany	RCT	G1 (Irradiation): 46 G2 (Diclofenac): 54	Control^c^: 100	G1: 52 G2: 48.1	31	G1: 65.9 G2: 63.9	65.3	6	6	0	0

^a^ Data derived from the population included in the primary outcome analysis (449 individuals across both groups). HO, was not the primary outcome of the study, and not all follow-up patients were evaluated for HO (Table 2).

^b^ Baseline data excluded patients who dropped out of the study. Groups are named from the second group, corresponding to the grouping in the authors’ study.

^c^ The control group was a historical control, as reported in another included study.

**TABLE 2:** Clinical interventions and outcomes.

Author/Ref	Diagnosis		Surgery and approach		Drug or irradiation intervention		Heterotopic ossification N°		Gastrointestinal side effects N°	
	Intervention	Control or placebo	Intervention	Control or placebo	Intervention	Control or placebo	Intervention	Control or placebo	Intervention	Control or placebo
Fransen et al. (2006)	Osteoarthritis (407) and others	Osteoarthritis (424) and others	THA (Anterior/anterolateral, Posterior/posterolateral and Others)	THA (Anterior/anterolateral, Posterior/posterolateral and Others)	Ibuprofen, 400 mg three times daily, 3 weeks	Placebo three times daily, 3 weeks	117 (391 checked) Brooker I: 78 Brooker II: 28 Brooker III: 9 Brooker IV: 2	177 (407 checked) Brooker I: 108 Brooker II: 43 Brooker III: 22 Brooker IV: 4	—	—
Persson et al. (1998)	Arthrosis	Arthrosis	THA (posterolateral approach)	THA (posterolateral approach)	G1: Ibuprofen, 400 mg three times daily, 3 weeks G2: Ibuprofen, 400 mg three times daily, 1 week; Placebo three times daily, 2 weeks	Placebo three times daily, 3 weeks	G1: 8 Brooker I: 7 Brooker II: 1 G2: 10 Brooker I: 6 Brooker II: 2 Brooker III: 2	21 Brooker I: 10 Brooker II: 4 Brooker III: 5 Brooker IV: 2	G1: 3 G2: 5	4
Ahrengart et al. (1994)	Arthrosis	Arthrosis	THA (Direct lateral approach)	THA (Direct lateral approach)	Ibuprofen, 500 mg three times daily, 10 days	Placebo three times daily, 10 days	14 (21 checked) Brooker I: 10 Brooker II: 3 Brooker III: 1	15 (26 checked) Brooker I: 6 Brooker II: 5 Brooker III: 3 Brooker IV: 1	6	0
Saudan et al. (2007)	Osteoarthritis (108) and others	Primary osteoarthritis (118) and others	THA	THA	G1:Celecoxib, 200 mg two times daily, 10 days G2: Ibuprofen, 400 mg two times daily, 10 days	None	G1: 48 (117 checked) Brooker I: 42 Brooker II-III: 6 G2: 73 (123 checked) Brooker I: 57 Brooker II-III: 16	None	G1: 1 G2: 3	None
Vielpeau et al. (1999)	Osteoarthritis	Osteoarthritis	THA (posterolateral/Lateral with trochanteric/Lateral without trochanteric)	THA (posterolateral/Lateral with trochanteric/Lateral without trochanteric)	G1: Naproxen, 250 mg three times daily, 6 weeks G2: Indomethacin, 25 mg three times daily, 6 weeks	Placebo, three times daily, 6 weeks	G1: 8 Brooker I: 6 Brooker II: 2 G2: 16 Brooker I: 14 Brooker II: 2	22 Brooker I: 10 Brooker II: 3 Brooker III: 9	G1: 6 G2: 5	7
Gebuhr et al. (1991)	Osteoarthritis	Osteoarthritis	THA (posterolateral approach)	THA (posterolateral approach)	Naproxen, 500 mg, twice on the day of surgery 250 mg three times daily, 4 weeks	Placebo, twice on the day of surgery three times daily, 4 weeks	4 Classification method reported by DeLee: I: 3 II: 1	15 (27 checked) Classification method reported by DeLee: I: 7 II: 5 III: 3	—	—
Reis et al. (1992)	Osteoarthritis (129) and others (All groups)		THA (transgluteal approach)<	THA (transgluteal approach)	Diclofenac, 50 mg three times daily, 6 weeks	Placebo, three times daily, 6 weeks	11 (74 checked) Classification method reported by DeLee: I: 8 II: 3	38 (69 checked) Classification method reported by DeLee: I: 7 II: 5 III: 3	11	3
Wahlstrom et al. (1991)	Arthrosis	Arthrosis	THA (dorsolateral approach)	THA (dorsolateral approach)	Diclofenac, 75 mg, twice on the day of surgery 50 mg three times daily, 6 weeks	Placebo, twice on the day of surgery three times daily, 6 weeks	1 (46 checked) <20 mm: 1	35 (47 checked) <20 mm: 2 ≥20 mm: 33	1	3
Sell et al. (1998)	G1: Osteoarthritis (56) and others G2: Osteoarthritis (51) and others	None	THA (transgluteal approach)	None	G1:Irradiation, 3 × 3.3 Gy G2: Diclofenac, 50 mg three times daily, 3 weeks	None	G1: 2 (76 checked) Brooker I: 2 G2: 18 (77 checked) Brooker I: 16 Brooker II: 2	None	G1:0 G2:11	None
Winkler et al. (2016)	G1: Osteoarthritis G2: Osteoarthritis	None	THA (lateral approach)	None	G1 (etoricoxib): Day −1 and 0 of surgery evening: 90 mg Day 1–7 of surgery morning: 90 mg evening: placebo G2 (diclofenac-sodium): Day −1 and 0 of surgery evening: 75 mg Day 1 until 7 of surgery morning: 75 mg evening: 75 mg	None	G1: 17 (45 checked) Brooker I: 13 Brooker II: 4 G2: 17 (44 checked) Brooker I: 12 Brooker II: 5	None	—	None
Kjaersgaard-Andersen et al. (1993)	Arthrosis (majority) and others	Arthrosis (majority) and others	THA (posterolateral approach)	THA (posterolateral approach)	Indomethacin, 25 mg three times daily, 2 weeks.	Placebo three times daily, 2 weeks	6 (Classification method reported by DeLee) I: 5 II: 1 III: 0	15 (Classification method reported by DeLee) I: 4 II: 5 III: 6	5	0
Kjaersgaard-Andersen et al. (1989)	Arthrosis	Arthrosis	THA (posterolateral approach)	THA (posterolateral approach)	Indomethacin, 25 mg three times daily, 6 weeks	Placebo three times daily, 6 weeks	12 (Classification method reported by DeLee) I: 12 II: 0 III: 0	62 (Classification method reported by DeLee) I: 18 II: 27 III: 17	—	—
Knelles et al. (1997)	G2: Arthritis (70) and others G3: Arthritis (77) and others G4: Arthritis (83) and others G5: Arthritis (65) and others G6: Arthritis (85) and others	Arthritis (74) and others	THA	THA	G2: Indomethacin, 50 mg two times daily, 14 days G3: Indomethacin, 50 mg two times daily, 7 days G4: Radiation, 4 × 3 Gy G5: Radiation, 1 × 7 Gy G6: Radiation, 1 × 5 Gy	Untreated	G2: 11 Brooker I: 8 Brooker II: 2 Brooker III: 1 G3: 18 Brooker I: 9 Brooker II: 7 Brooker III: 2 G4: 5 Brooker I: 5 G5: 11 Brooker I: 11 G6: 28 Brooker I: 23 Brooker II: 4 Brooker III: 1	65 Brooker I: 26 Brooker II: 15 Brooker III: 19 Brooker IV: 5	G2: 4 G3: 5 G4: 0 G5: 0 G6: 0	0
Kienapfel et al. (1999)	G1: Osteoarthritis (32) and others G2: Osteoarthritis (35) and others	Osteoarthritis (31) and others	THA (lateral transgluteal approach)	THA (lateral transgluteal approach)	G1: Radiation, 1 × 600 cGy G2: Indomethacin, 50 mg two times daily, 6 weeks	Untreated	G1: 12 Brooker I: 10 Brooker II: 2 G2: 20 Brooker I: 17 Brooker II: 3	G1: 30 Brooker I: 8 Brooker II: 9 Brooker III: 11 Brooker IV: 2	G1: 4 G2: 15	5
Bremen-Kuhne et al. (1997)	Arthrosis (majority) and others	None	THA	None	G1: Radiation, a single dose of 6 Gy G2: Indomethacin, 100 mg (suppo.) on the day of surgery, 75 mg once daily for 1–10 days after surgery	None	G1: 9 (19 checked) Brooker I-II:9 G2: 11 (31 checked) Brooker I-II: 10 Brooker III-IV: 1	None	—	None
van Leeuwen et al. (1998)	Arthrosis (majority) and others	Arthrosis (majority) and others	THA (posterolateral approach	THA (posterolateral approach)	Radiation, a single dose of 6 Gy, the day before the surgery	Untreated	6 (43 checked, 2 of the 41 had two surgeries) Brooker I: 5 Brooker III: 1	16 (19 checked, 3 of the 16 had two surgeries) Brooker I: 4 Brooker II: 4 Brooker III: 5 Brooker IV: 3	—	—
Kolbl et al. (1998)	G1: Coxarthrosis (42) and others G2: Coxarthrosis (44) and others	Coxarthrosis (74) and others	THA	THA	G1: Irradiation with single 7 Gy fraction within 16–20 h before operation. G2: Diclofenac, 75 mg two times daily, 2 weeks	Untreated	G1: 22 (46 checked) Brooker I: 17 Brooker II: 4 Brooker III: 1 G2: 6 (54 checked) Brooker I: 5 Brooker II: 1	65 (100 checked) Brooker I: 26 Brooker II: 15 Brooker III: 19 Brooker IV: 5	G1: 0 G2: 3	0

### Model convergence

**FIGURE 3:**
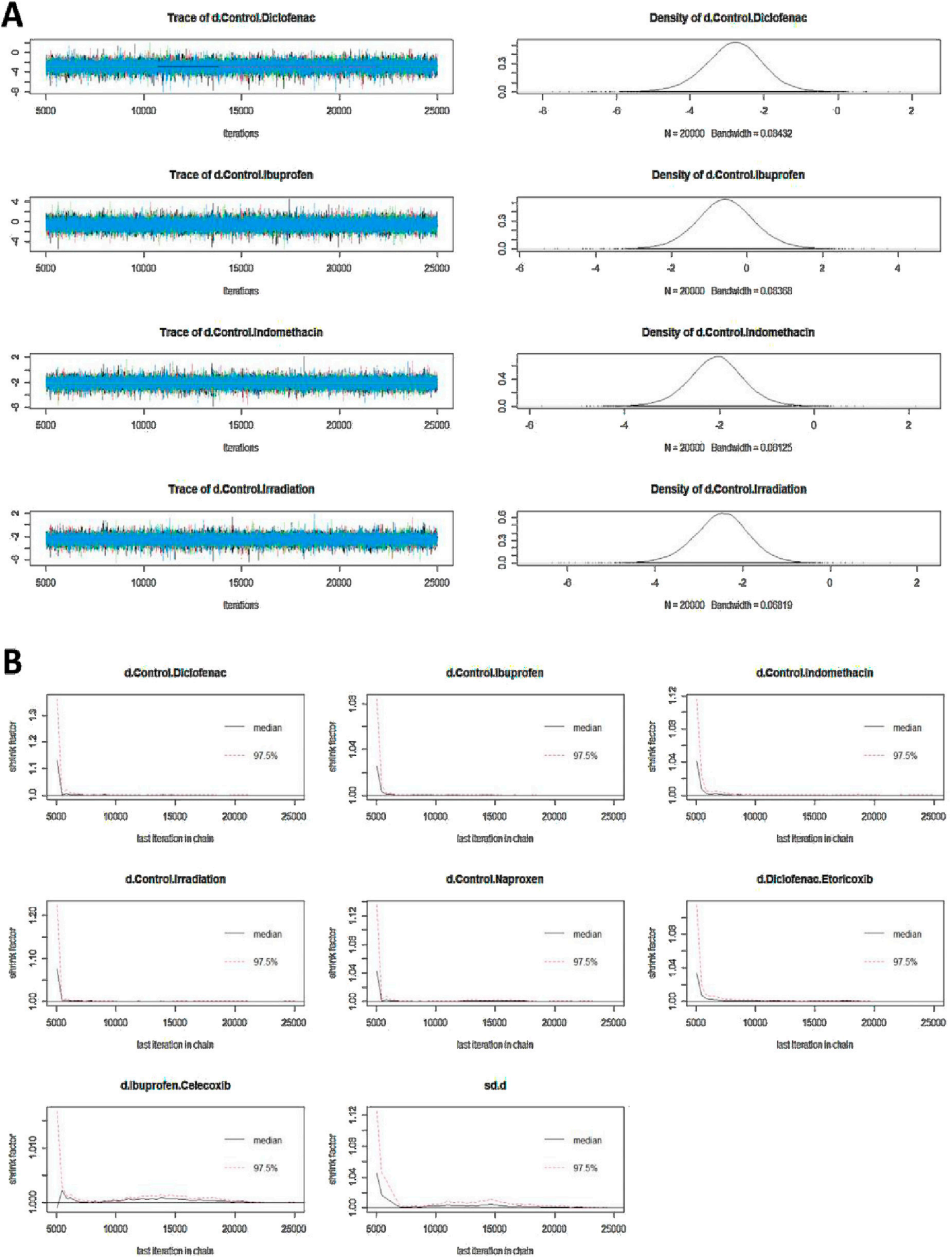
**(A)** Trajectory plot and density plot. **(B)** Brooks-Gelman-Rubin diagnostic plot.

The trajectory plot in Figure 3A indicates that fluctuations tend to stabilize when the number of iterations exceeds 5,000; by 20,000 iterations, the density map shows a normal distribution, and the bandwidth narrows to 0, indicating stability. Figure 3B, the Brooks-Gelman-Rubin diagnostic plot, shows that the median and 97.5% of the potential scale reduction factor (PSRF) tend to 1, achieving stability after iterative calculation. The PSRF value is close to 1 (Table 3), suggesting that the model has converged satisfactorily.

**TABLE 3:** Potential scale reduction factor (PSRF).

	Point est.	Upper C.I.
d.Control.Diclofenac	1	1
d.Control.Ibuprofen	1	1
d.Control.Indomethacin	1	1
d.Control.Irradiation	1	1
d.Control.Naproxen	1	1
d.Diclofenac.Etoricoxib	1	1
d.Ibuprofen.Celecoxib	1	1
sd.d	1	1

### Incidence of HO

**FIGURE 4:**
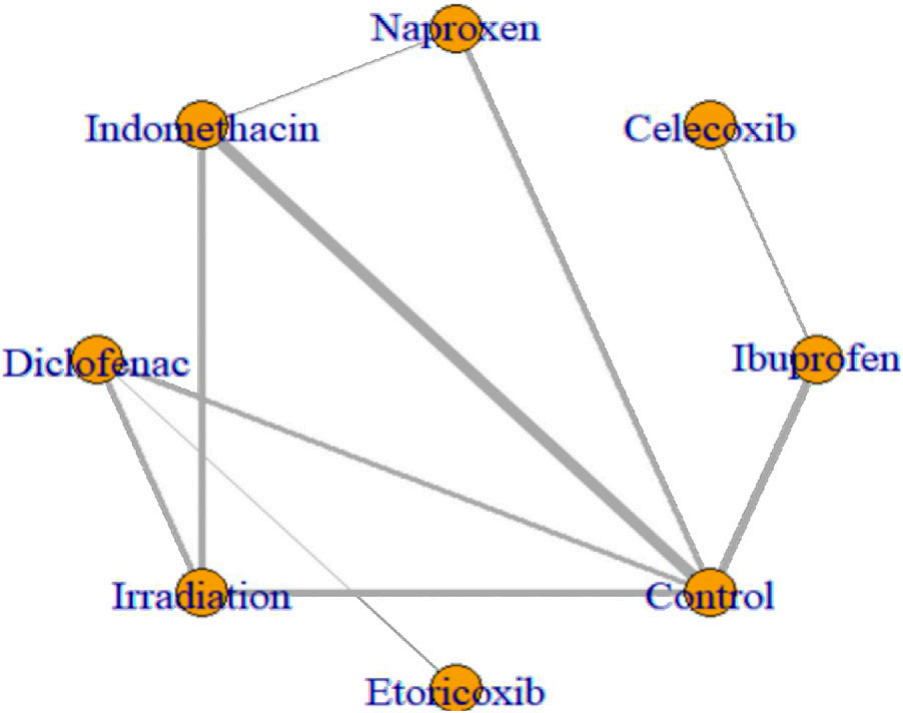
Network plots of eligible comparisons of HO. Nodes represent different ways to intervene. The connection line shows a direct comparison between the two ways to intervene. The thickness of the lines shows the sample size for the direct comparison of the two ways to intervene.

**FIGURE 5:**
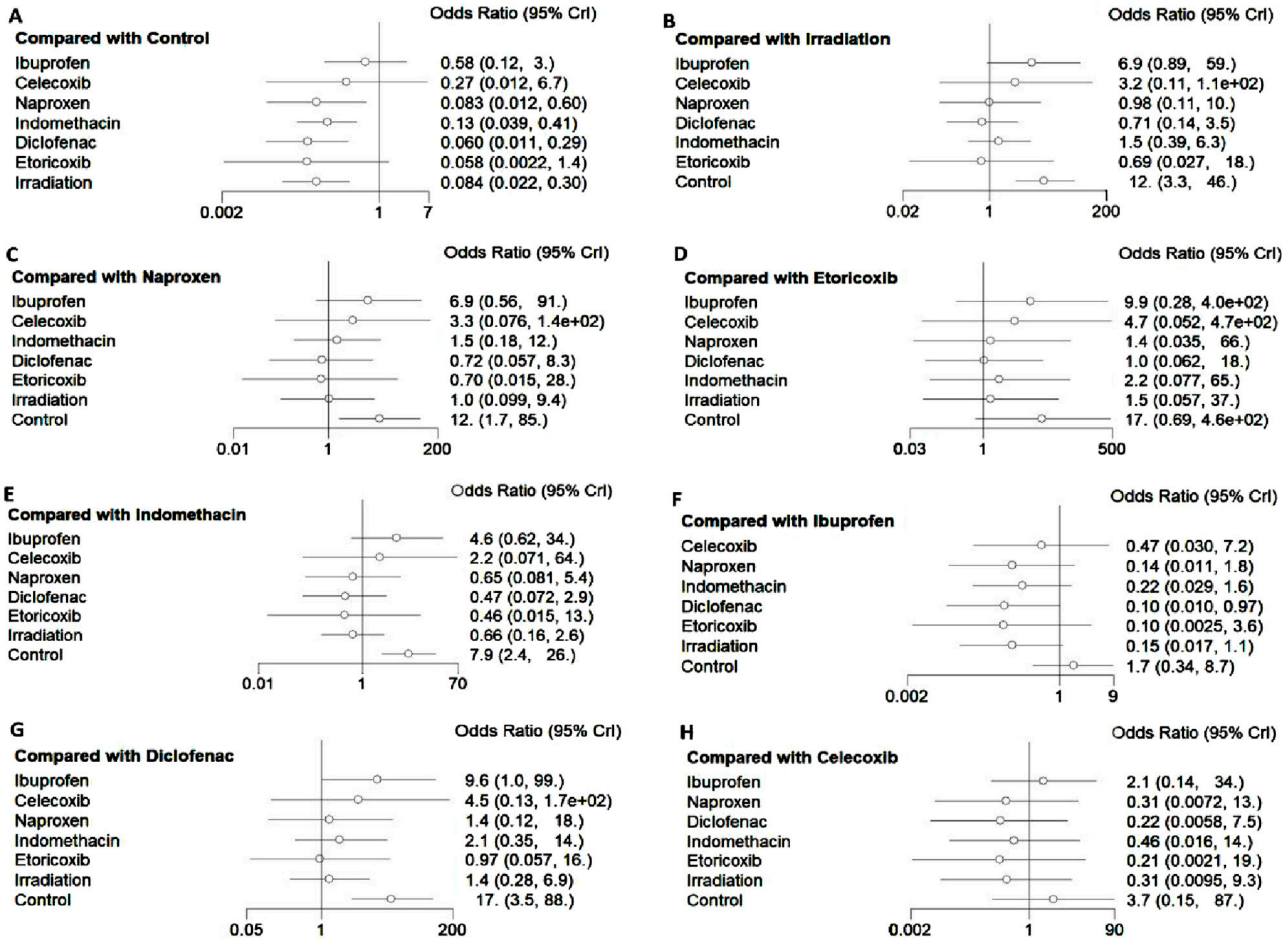
Impact of different interventions on HO after THA. Data are presented as odds ratios (OR) and 95% credible intervals ((95% CrI). **(A)** Efficacy of different interventions in HO compared with the control group, **(B)** Efficacy of different interventions in HO compared with irradiation, **(C)** Efficacy of different interventions in HO compared with naproxen, **(D)** Efficacy of different interventions in HO compared with etoricoxib, **(E)** Efficacy of different interventions in HO compared with indomethacin, **(F)** Efficacy of different interventions in HO compared with ibuprofen, **(G)** Efficacy of different interventions in HO compared with diclofenac, **(H)** Efficacy of different interventions in HO compared with celecoxib.

**FIGURE 6:**
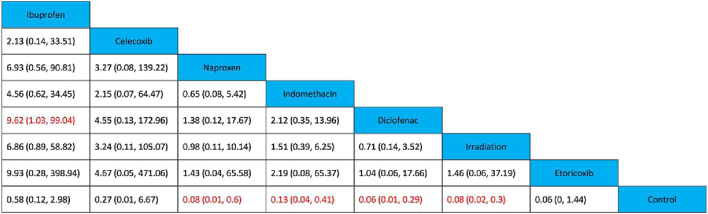
Relative effects of various strategies on HO after THA. Data are presented as OR and 95% credible intervals, with significant differences highlighted in red.

All 17 studies reported the incidence of HO, and the eligible comparisons among them are presented in a network diagram (Figure 4). The total incidence rate of HO in all control groups was 55.2%. Figure 5 is a forest plot illustrating the incidence of HO following various interventions, highlighting their effectiveness in HO prevention. Figure 6 shows the relative effects of different strategies for HO prevention, expressed as odds ratios (ORs) rounded to two decimal places. In terms of effectiveness, compared with the control, four strategies showed a low incidence of HO, including naproxen (OR = 0.08, 95% CrI 0.01–0.60), indomethacin (OR = 0.13, 95% CrI 0.04–0.41), diclofenac (OR = 0.06, 95% CrI 0.01–0.29), and irradiation (OR = 0.08, 95% CrI 0.02–0.3) (Figures 5A, 6). Diclofenac was more beneficial than ibuprofen (OR = 0.10, 95% CrI 0.01–0.97) as indicated in Figure 5F. Pairwise comparisons of the other four drugs, as well as comparisons between irradiation and all drugs, showed no significant differences.

Seventeen studies were included for direct or indirect comparisons. Incidences of HO after THA were independently compared for each intervention; ORs and corresponding 95% CrIs were calculated. Concerning the efficacy of drugs versus irradiation, the six NSAIDs did not demonstrate clear advantages. The Brooker classification, the most commonly used method to assess the severity of HO, was employed in 11 of our 17 studies. Given that severe HO can cause clinical symptoms, we also gathered data on the incidence of Brooker grades III–IV (Table 4). According to the data, 13.48% of the control group developed Brooker grade III–IV HO. Nevertheless, in the intervention group, the incidence of Brooker grade III–IV HO was only 2.77% in the ibuprofen group, followed by 1.58% in the indomethacin group. Due to the significant differences in the criteria for reporting complications in various studies, we have summarized the most critical and significant gastrointestinal side effects. Information on gastrointestinal side effects was compiled from 12 studies that explicitly reported these occurrences. We collected data on the incidence of gastrointestinal side effects (Table 4). According to the data, the incidence of gastrointestinal side effects was as follows: Naproxen (23.08%), indomethacin (8.19%), ibuprofen (7.14%), irradiation (5.22%), diclofenac (5.08%), control (3.26%), celecoxib (0.85%), and etoricoxib (0.00%). From the above results it is easy to see that the incidence of gastrointestinal side effects of selective COX-2 inhibitors is lower than that of non-selective COX-2 inhibitors, but unfortunately most of the included studies did not report the incidence of cardiovascular and cerebrovascular events in detail. We had intended to conduct a meta-analysis on Brooker III-IV HO and gastrointestinal side effects; however, due to limited data, substantial heterogeneity, and failure to pass the consistency test, this was not feasible.

**TABLE 4:** Incidence of Brooker III-IV HO and incidence of gastrointestinal side effects by intervention.

Intervention	Patients N°	Brooker III-IV HO N°	Author/Ref	Patients N°	Gastrointestinal side effects N°	Author/Ref
Ibuprofen	506	14	Persson et al. (1998) Fransen et al. (2006) Ahrengart et al. (1994)	238	17	Persson et al. (1998) Ahrengart et al. (1994) Saudan et al. (2007)
Naproxen	26	0	Vielpeau et al. (1999)	26	6	Vielpeau et al. (1999)
Indomethacin	317	5	Vielpeau et al. (1999) Knelles et al. (1997) Kienapfel et al. (1999) “Bremen-Kuhne et al. (1997)”	305	25	Vielpeau et al. (1999) Kjaersgaard-Andersen et al. (1993) Knelles et al. (1997) Kienapfel et al. (1999)
Diclofenac	121	0	Sell et al. (1998) Winkler et al. (2016) Kolbl et al. (1998)	295	15	Reis et al. (1992) Wahlstrom et al. (1991) Sell et al. (1998) Winkler et al. (2016) Kolbl et al. (1998)
Irradiation	522	0	Sell et al. (1998) Knelles et al. (1997) Kienapfel et al. (1999) “Bremen-Kuhne et al. (1997)” van Leeuwen et al. (1998) Kolbl et al. (1998)	460	24	Sell et al. (1998) Knelles et al. (1997) Kienapfel et al. (1999) Kolbl et al. (1998)
Etoricoxib	45	0	Winkler et al. (2016)	45	0	Winkler et al. (2016)
Celecoxib	—	—	—	117	1	Saudan et al. (2007)
Control	675	91	Persson et al. (1998) Fransen et al. (2006) Ahrengart et al. (1994) Vielpeau et al. (1999) Knelles et al. (1997) Kienapfel et al. (1999) van Leeuwen et al. (1998) Kolbl et al. (1998)	675	22	Persson et al. (1998) Ahrengart et al. (1994) Vielpeau et al. (1999) Reis et al. (1992) Wahlstrom et al. (1991) Kjaersgaard-Andersen et al. (1993) Knelles et al. (1997) Kienapfel et al. (1999) Kolbl et al. (1998)

### Ranking of interventions

**FIGURE 7:**
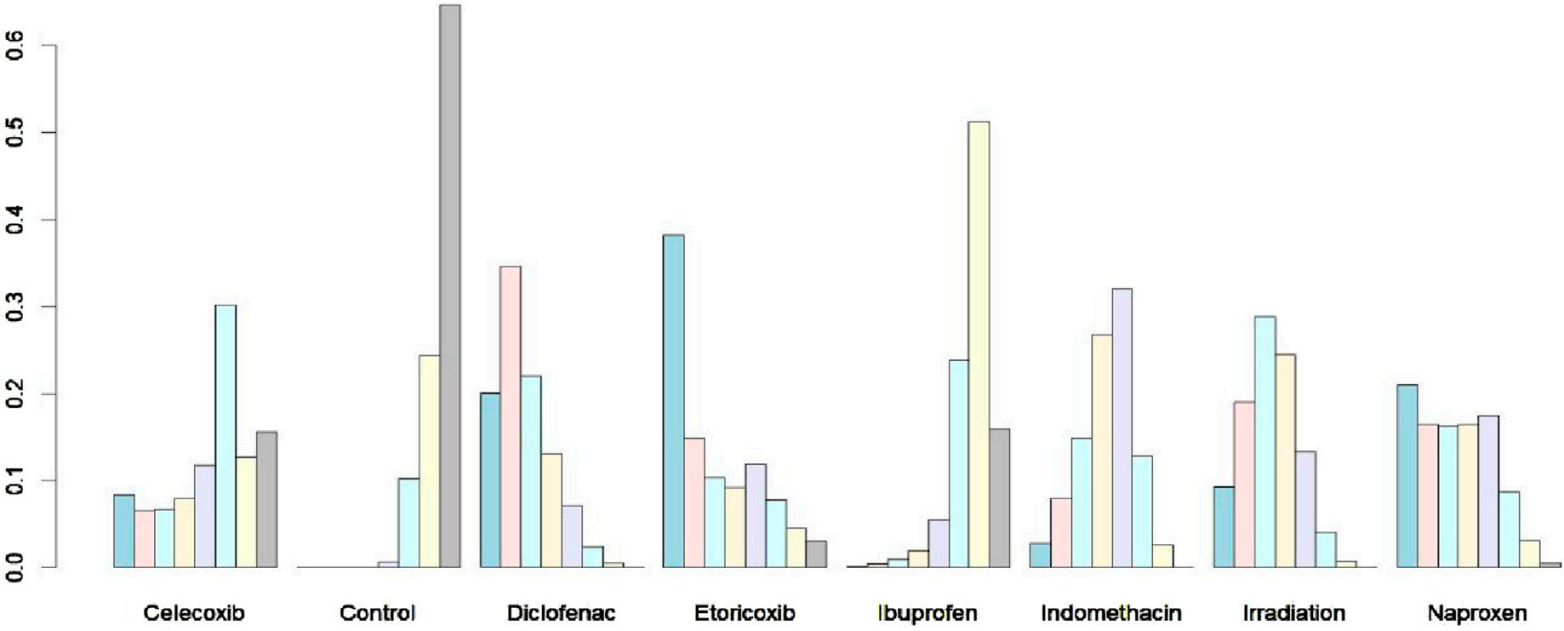
Rank probability of different strategies. The probability of each of the intervention measures at different positions in the ranking.

The rank probability plot (Figure 7) illustrates the ranking probabilities for seven interventions and controls. Etoricoxib is shown to have the highest probability as the preferred option, while ibuprofen is the least effective among all interventions. The probabilities derived from the surface under the cumulative ranking curve (SUCRA) algorithm are as follows: Diclofenac 78.0%, Etoricoxib 71.6%, Irradiation 67.3%, Naproxen 66.7%, Indomethacin: 53.2%, Celecoxib, 38.8%, Ibuprofen 18.6%, and Control 6.8%. Although diclofenac and etoricoxib occupy different positions, they have demonstrated consistent effects in only one randomized controlled trial. However, stronger evidence supports the efficacy of diclofenac. The most likely order of effectiveness is: Diclofenac > Etoricoxib > Irradiation > Naproxen > Indomethacin > Celecoxib > Ibuprofen; it is noteworthy that etoricoxib and diclofenac have shown equivalent effects in the study.

### Consistency check

**FIGURE 8:**
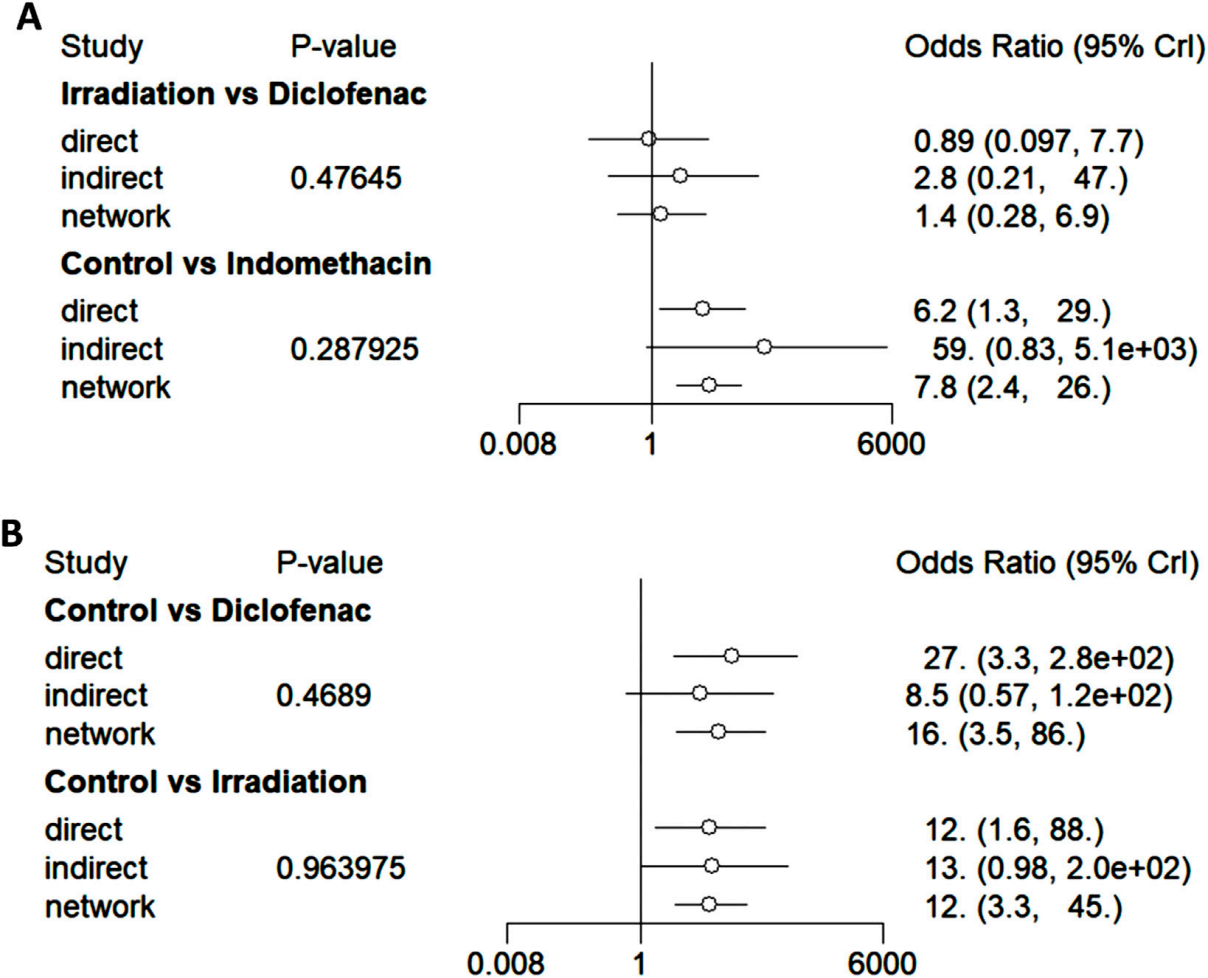
Forest plots of consistency (node-splitting model). **(A)** The forest plots of consistency tests for irradiation versus diclofenac and control versus indomethacin. **(B)** The forest plots of consistency tests for control versus diclofenac and control versus irradiation.

The node-splitting model was employed to perform a consistency test (Figure 8). Among the direct and indirect comparisons, as well as network comparison results of the interventions between irradiation and diclofenac, control and indomethacin, control and diclofenac, and control and irradiation, the *P*-values are all greater than 0.05, indicating good consistency.

### Heterogeneity and sensitivity analysis

**FIGURE 9:**
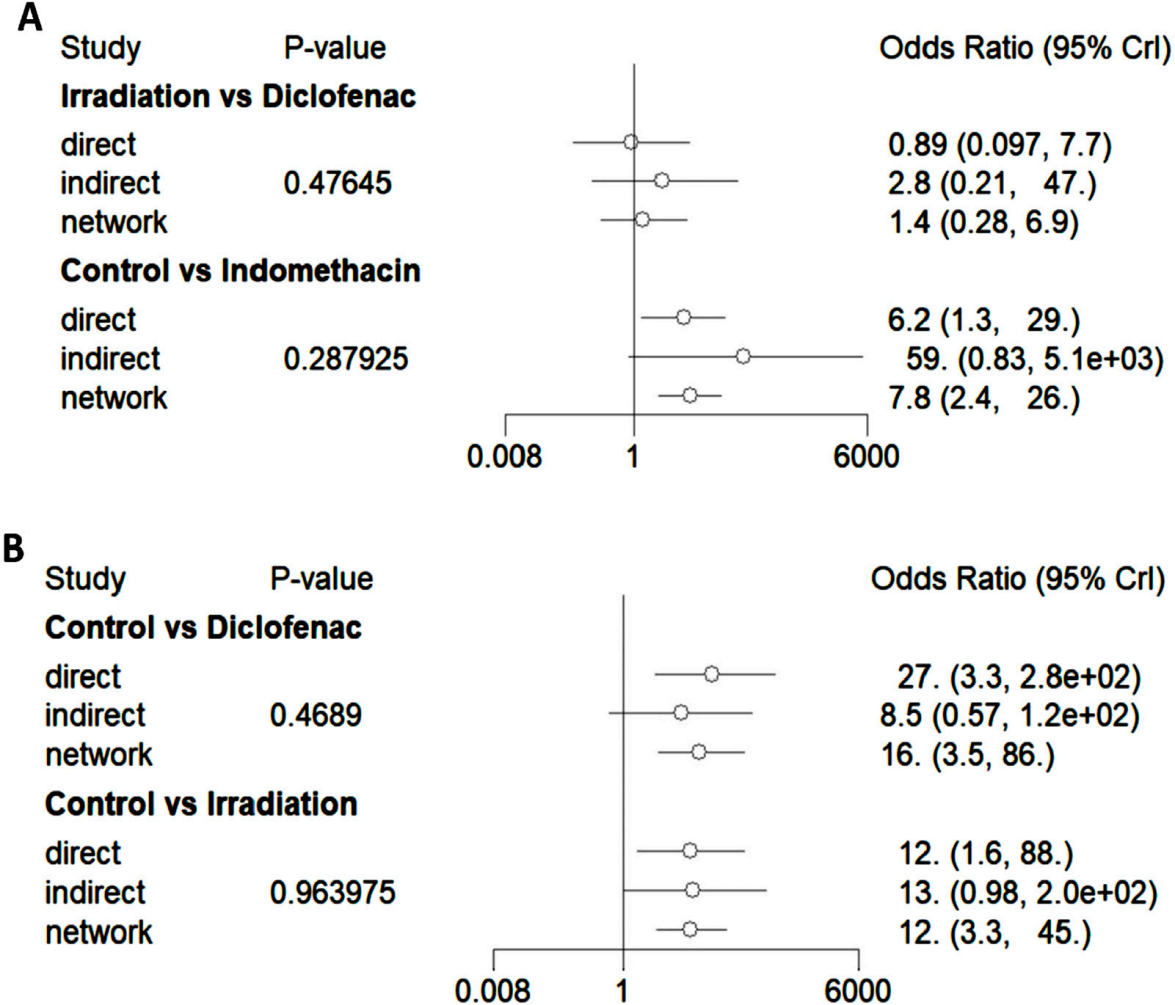
Forest plots of heterogeneity test. **(A)** The results of heterogeneity test for irradiation versus diclofenac and control versus indomethacin. **(B)** The results of heterogeneity test for control versus diclofenac and control versus irradiation.

**FIGURE 10:**
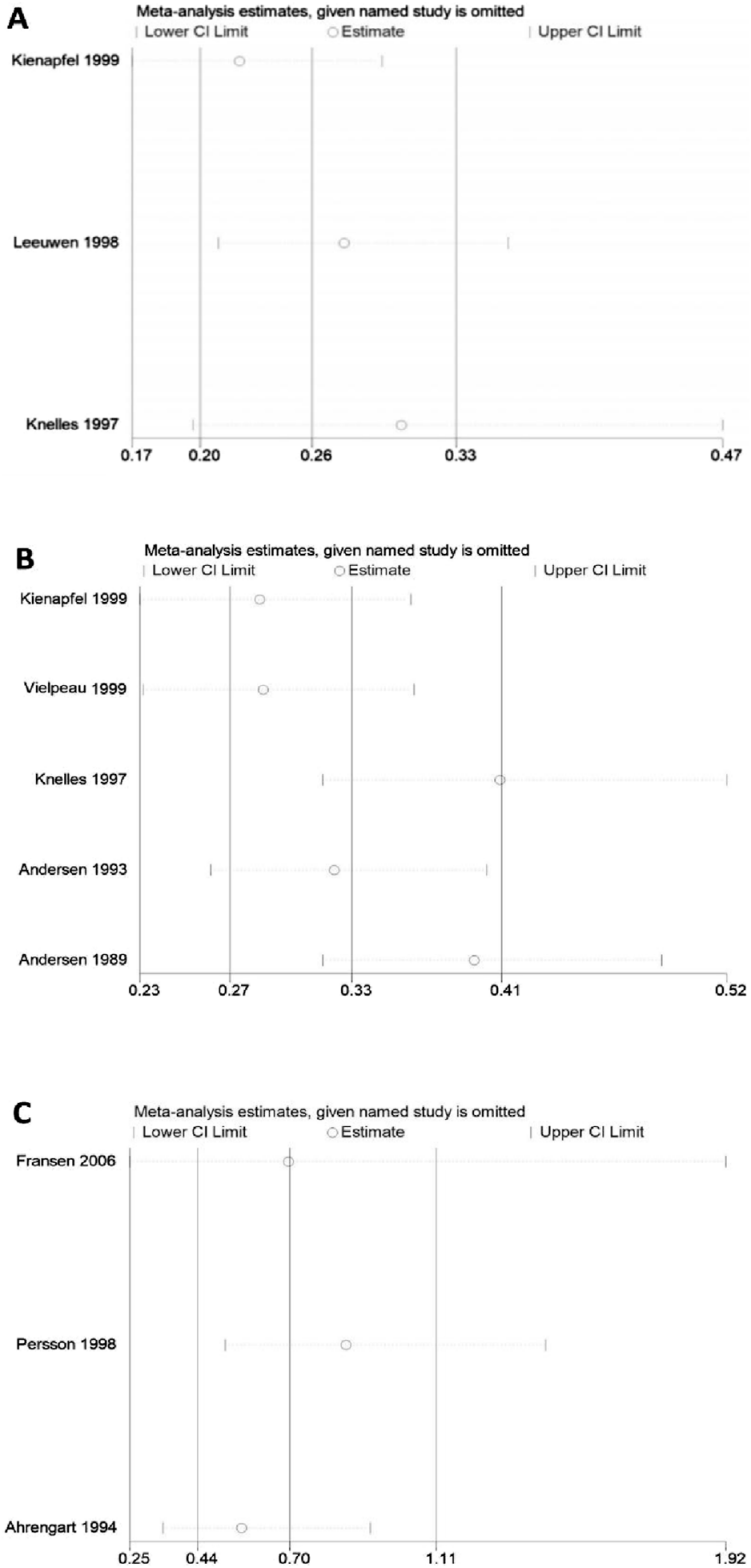
Sensitivity analysis of HO **(A)** Irradiation. **(B)** Indomethacin. **(C)** Ibuprofen.

No significant heterogeneity was observed in pairwise comparisons among five groups. Direct comparisons among the other five groups showed significant heterogeneity (Figure 9), including: direct comparison of diclofenac and control (91.8%), ibuprofen and control (67%), and indomethacin and control, as well as irradiation versus diclofenac (95.9%), and RT versus control, which showed moderate heterogeneity (59.4%). Comparisons with substantial heterogeneity that included at least three studies were further subjected to sensitivity analysis using the one-by-one elimination method (Figure 10). Only two studies were included in the remaining two comparisons, and their heterogeneity was directly analyzed. The results confirm the stability of the meta-analyses for indomethacin versus control and diclofenac versus control. The figure shows that the results of the direct comparison between ibuprofen and control are not robust enough. The results of direct and indirect comparisons of diclofenac and control, indomethacin and control, irradiation and control, and irradiation and diclofenac were consistent.

### Small-study effect

**FIGURE 11:**
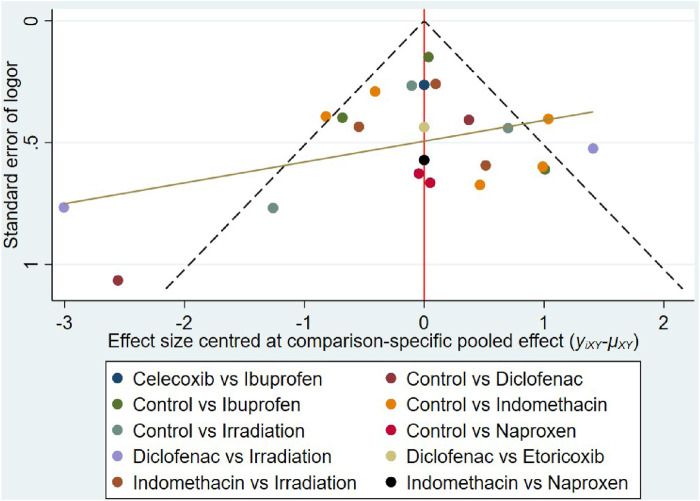
Funnel plots of the network comparisons.

The funnel plot shows asymmetry (Figure 11), indicating the presence of a small sample effect in this study.

## Discussion

The prevention of HO after THA has always been a significant concern (Wang et al., 2023b; Wang et al., 2023c). Consistent with prior studies, our findings indicate that among 926 cases in the control group, the incidence of HO was 55.2% (Herzberg et al., 2024), with 13.48% Of the 675 control cases evaluated using the Brooker scale developed grade III–IV HO. However, for all interventions, the incidence of Brooker grade III–IV HO was lower in the intervention group than in the control group. These findings suggest that both non-steroidal anti-inflammatory drugs and irradiation intervention have a beneficial effect in preventing HO after THA.

The heterogeneity in the direct comparison between diclofenac and control arises from variations in study sample sizes and the proportion of HO occurrence; however, both studies affirm diclofenac’s effectiveness in preventing HO after THA. This consistency bolsters our meta-analysis. Reis et al. reported that oral administration of diclofenac for 6 weeks reduced HO from 55% to 15% compared to placebo (Reis et al., 1992). Wahlstrom et al.’s study demonstrated that diclofenac completely prevented significant bone formation with minimal complications (Wahlström et al., 1991). A prospective study of 644 patients with THO reported similar results: 80% of patients in the diclofenac group did not develop ossification, and none developed Brooker grades Ⅳ HO (Jockheck et al., 1998). Sell et al. compared the incidence of HO following 2-week treatments with 150 mg and 75 mg of diclofenac per day, finding no significant difference between the two groups 6 months post-treatment, which supports a short-term, lower-dose treatment regimen (Sell et al., 1998). Haffer advocated a clear protocol, recommending the daily administration of at least 75 mg of diclofenac from the first day post-surgery for at least 9 days to effectively prevent HO (Haffer et al., 2022). Stable results from comparisons of diclofenac to irradiation further underscore diclofenac’s superiority (K̈olbl et al., 1998).

Etoricoxib, a selective cyclo-oxygenase-2 (COX-2) inhibitor, is likely ranked second in the comprehensive ranking. In the only study, etoricoxib demonstrated a similar HO incidence rate to diclofenac (17/45, 17/44), but etoricoxib had significantly fewer complications. As noted by Oberberg et al. in a study involving 194 participants, etoricoxib can effectively prevent HO after primary THA, offering a lower complication rate (Oberberg et al., 2021). A smaller study found Etoricoxib and indomethacin to have comparable preventive effects against HO. Among patients treated with etoricoxib, 31% developed Brooker grades I and II ossification, with 7% developing ossification, and no severe ossification was reported (Brunnekreef et al., 2013). Given the minor differences between the two drugs in our study and the scarcity of published studies, further research is warranted to provide a definitive comparison.

As a classic preventive measure against HO, local irradiation effectively reduces the occurrence of HO after THA (Heyd et al., 1995; Kruser et al., 2012). Both direct and indirect comparisons in our meta-analysis demonstrated the effectiveness of irradiation. We attribute the main cause of heterogeneity to sample bias across studies, but consistent preferences for irradiation were observed, and sensitivity analysis supports the robustness of our results. Controversy exists regarding the timing and dose of irradiation; some studies have employed postoperative fractionated irradiation. Kolbl et al. compared a single postoperative dose of 5 Gy with 7 Gy and found the latter to be an effective preventive regimen (K̈olbl et al., 1998). Conversely, numerous studies support the efficacy of low-dose and single-shot irradiation for preventing HO. Seegenschmiedt et al. found no difference in HO incidence between two doses of 5 × 2 Gy and 10 × 2 Gy (Seegenschmiedt et al., 1993). Lo et al. determined that a single postoperative dose of 700-cGy yielded results equivalent to fractionated irradiation (Lo et al., 1988), a finding corroborated by Hedley et al. (1989) and Healy et al. (1990). Heyd et al. noted good outcomes with preoperative irradiation, positing it as an alternative to postoperative irradiation (Heyd et al., 1997). A study involving 462 hips reported effective treatment with preoperative irradiation the night before surgery (Koelbl et al., 2003). Another study concurred, suggesting that patient management could be simplified and complications related to postoperative transportation reduced (Seegenschmiedt et al., 1994). Gregoritch et al. recommended irradiation within 4 h before surgery for satisfactory results (Gregoritch et al., 1994).

Concerns about radiation potentially causing loosening of joint prostheses or tumor occurrence have been addressed by several studies. Pakos et al. followed 97 patients (with three fatalities during follow-up) for 10 years and observed no tumors or prosthesis loosening in the irradiation and indomethacin group, similar to outcomes in the control group treated with indomethacin alone (Pakos et al., 2020). Significant heterogeneity was noted in comparisons between irradiation and diclofenac, attributable to disparate findings in the included studies. Kolbl et al. reported HO in 22 of 46 patients in the irradiation group compared to 6 of 54 in the diclofenac group (K̈olbl et al., 1998). In contrast, Sell et al. observed HO in 2 of 76 patients in the irradiation group and 18 of 77 in the diclofenac group (Sell et al., 1998), possibly due to differences in the timing and dosage of administered irradiation (the former study used a single dose before surgery, while the latter used three post-surgery doses). This calls for more robust studies to clarify the efficacy of diclofenac versus irradiation.

Studies on the priority of naproxen in HO prevention after hip surgery support its effectiveness. In a randomized controlled trial, Gebuhr et al. administered 250 mg of naproxen three times daily for 4 weeks, significantly reducing the incidence of ectopic ossification after bone cement THA (Gebuhr et al., 1991). Vielpeau et al. found that the same dosage over 6 weeks was more effective than indomethacin and placebo (Vielpeau et al., 1999). A meta-analysis involving 269 patients indicated that naproxen was associated with significantly lower HO rates after hip surgery, including arthroscopic surgery (Ma et al., 2018). Another study corroborated these findings (Zhang et al., 2019). Macfarlane et al. reported that naproxen and diclofenac, which are equally effective as indomethacin, can be considered as first-line treatment for the prevention of HO after THA (Macfarlane et al., 2008).

Among the drugs studied, indomethacin was most frequently included and once regarded as the “gold standard” for the pharmacological prevention of HO (Macfarlane et al., 2008). A previous systematic review reported indomethacin as the most effective drug available at the time (Fijn et al., 2003). Both the effectiveness and safety of indomethacin have been confirmed (Tözün et al., 1992), and our meta-analysis corroborates these findings. Several studies have demonstrated that a 2-week regimen of indomethacin can effectively reduce the incidence of HO (Hofmann et al., 1999). Wuning et al. compared 100 mg of indomethacin daily over 7 days with a 14-day regimen, finding similar benefits between the two durations (Wurnig et al., 1997). However, shorter indomethacin courses were not more beneficial; Heide et al. reported that a 3-day regimen was ineffective in preventing HO post-THA (van der Heide et al., 2007). Dorn et al. found an 8-day course more effective than a 4-day course (Dorn et al., 1998). Another study indicated that a 5-day course failed to achieve the desired preventive effect (Koorevaar et al., 1999). Both short-term indomethacin treatment and single-dose irradiation of 6 Gy have been shown to reliably prevent severe HO, with no significant difference in effectiveness between the two (Bremen-Kuhne et al., 1997). Burd et al. reported no differential effect in preventing HO after acetabular fracture surgery between these approaches (Burd et al., 2001).

As an early selective COX-2 inhibitor, celecoxib has increasingly been preferred by physicians due to its low risk of gastrointestinal complications. One study involving 150 participants in celecoxib and 250 participants in indomethacin groups showed equivalent efficacy in preventing periarticular ossification post-hip replacement, with the celecoxib group experiencing significantly fewer adverse reactions (Romanò et al., 2004). Only one celecoxib study met the inclusion criteria: it included 250 patients treated with oral ibuprofen or celecoxib for 10 days, revealing that 50% of the patients in the ibuprofen group developed ectopic ossification, compared to 41% in the celecoxib group (Saudan et al., 2007). A multicenter prospective observational study following 480 patients for 12 months reported a 20% incidence of HO in those treated with celecoxib, with no cases of Brooker grade 3 or 4; the incidence of HO in untreated patients was 55%, and 8.9% were Brooker grade 3 or 4 (Barbato et al., 2012). Another retrospective study observed that 14 of 106 patients treated with celecoxib developed HO, 11 of whom were Brooker grades 3 or 4, compared with 35 of 188 who had HO, 26 of whom were Brooker grades 3 or 4 (Oni et al., 2014). Lavernia et al. also endorse celecoxib as an effective and safe preventive measure for HO post-THA (Lavernia et al., 2014). The results of this study show that the incidence of gastrointestinal side effects of selective COX-2 inhibitors is lower than that of non-selective COX-2 inhibitors. In addition, recent expert opinion also recommends selective COX-2 inhibitors, such as celecoxib and etoricoxib, as the first choice for preventing HO in patients with a first THA, but a comprehensive assessment of the patient’s condition and vigilance for cardiovascular events are needed. (Migliorini et al., 2022). Nevertheless, given the limited number of studies on celecoxib, we recommend conducting more randomized controlled trials to strengthen these findings.

What is surprising is that the analysis results do not support the effectiveness of ibuprofen in preventing HO. The heterogeneity of this analysis stems from the large disparities in sample sizes across three studies and their contradictory conclusions. Based on this research, the effectiveness of ibuprofen in preventing HO remains uncertain, and it ranked last in our meta-analysis. This outcome aligns with previous studies that also report inconsistencies regarding ibuprofen. Koorevaar observed 95 patients and noted that a 5-day ibuprofen regimen failed to prevent post-THA HO (Koorevaar et al., 1999). A comparative study concluded that daily oral administration of 100 mg of ibuprofen for 3 weeks was comparable to indomethacin for post-THA prophylaxis (Schneider et al., 2023). However, the indomethacin group was treated from 2017 to 2019, while the ibuprofen group was treated from 2019 to 2022, introducing potential bias. Although it appeared that fewer people in the ibuprofen group developed HO, our meta-analysis revealed no significant difference between ibuprofen and the control group. This contrasts with a previous meta-analysis that found ibuprofen generally effective in reducing HO incidence post-THA (Tariq et al., 2023). The discrepancy arises because one favorable study on ibuprofen was excluded based on our criteria, where one-third of patients in the ibuprofen group developed HO, compared to three-quarters in the placebo group. Fijn et al. also suggest that short-term ibuprofen use is ineffective (Elmstedt et al., 1985).

Since only one randomized controlled trial comparing ibuprofen with celecoxib met the inclusion criteria, the incidence of HO was lower in the celecoxib group than in the ibuprofen group (48/117 vs. 73/123). Celecoxib ranked only ahead of ibuprofen in our network analysis, which may be influenced by the potential ineffectiveness of ibuprofen.

In general, our research supports the effectiveness of diclofenac, irradiation, naproxen, and indomethacin in preventing HO after THA, while the efficacy of ibuprofen remains unconfirmed. Concerning the lower incidence of complications associated with celecoxib and etoricoxib, these drugs may hold promise, and we advocate for further studies to verify their benefits. Mild (Brooker grade I) to moderate (Brooker grade II) HO does not impair pain sensation or hip function (Slätis et al., 1978), hence our focus is primarily on interventions effective in reducing severe (Brooker grade III-IV) HO occurrences. Unfortunately, current data are insufficient for a NMA on this matter. Special attention must also be given to preventing gastrointestinal side effects when using NSAIDs. Despite failing to conduct a meta-analysis on this aspect, we are concerned that gastrointestinal side effects were the most common reason participants withdrawal from studies, even though some studies used gastric protective drugs (Knelles et al., 1997; Kienapfel et al., 1999).

The occurrence of HO is multidimensional, and its clinical course is significantly regulated by surgical technique, gender and comorbidities, in addition to pharmacological interventions and radiation therapy (Wang et al., 2024). A series of studies have demonstrated a significant correlation between the choice of surgical approach for THA and the occurrence of HO. The incidence of HO after modified anterolateral approach and STD-Bauer approach was found to be significantly higher than that of minimally invasive surgical routes such as direct anterior approach (DA) (Hürlimann et al., 2017). Furthermore, a retrospective study confirmed that the incidence of HO in the group of DA approach (19.4%) was significantly lower than that in the group of direct posterolateral approach (36.1%) (Alijanipour et al., 2017). This phenomenon may be closely related to minimally invasive surgery for the extent of soft tissue stripping and minimisation of trauma. Furthermore, comprehensive measures such as meticulous intraoperative removal of bone debris, avoidance of cement fixation and reduction of local haematoma formation have been shown to reduce the risk of HO (Kantak and Shah, 2017; Johnson et al., 2025). Gender as an independent variable of HO occurrence: although the incidence of postoperative HO is slightly higher in male patients than in females, the available evidence is not yet sufficient to support a gender-stratified management strategy (Aprato et al., 2023). A review of the extant literature on comorbidity-related studies indicated that patients with a history of previous HO, bilateral proliferative osteoarthritis (men), ankylosing spondylitis, diffuse idiopathic osteomalacia, and Paget’s disease constituted a high-risk group for postoperative HO after THA (Iorio and Healy, 2002). Nevertheless, the study was unable to analyse the subgroups in depth due to the incompleteness of the sample data. It is important to note that HO prophylaxis must adhere to the principle of multimodal management, encompassing preoperative risk assessment, intraoperative minimally invasive procedures, and postoperative dynamic management. The present study employed a net meta-analysis to compare the different effects of six commonly used NSAIDs as well as irradiation in HO prophylaxis after THA, with the aim of providing some references for the development of clinical strategies.

### Limitations

Firstly, our analysis encompassed only a limited number of studies, and recent reports have not included any studies that met the specified criteria. This limitation may potentially impact the timeliness and generalizability of the findings in this article. Despite incorporating the latest research, as illustrated in the final funnel plot, a risk of publication bias persists; secondly, a lack of sufficient evidence and incomplete comparisons hinder forming a closed loop, potentially biasing indirect comparisons. Additionally, the occurrence of heterotopic ossification (HO) is influenced by a range of risk factors, including surgical approach, use of bone cement, intraoperative irrigation, and complications. Due to limitations in the available data, we were unable to conduct an analysis comparing differences among types of prostheses and surgical approaches. This limitation may potentially affect the comprehensiveness of the results. Finally, insufficient data prevented comparison of severe Brooker grades and complications, resulting in an inadequate assessment of drug safety.

## Conclusion

In terms of preventive efficacy, diclofenac and etoricoxib demonstrated the most favorable performance in preventing HO after THA within this network meta-analysis. Irradiation, naproxen, and indomethacin are also satisfactory options, while ibuprofen is ineffective. Given the advantages shown by etoricoxib and celecoxib, further randomized controlled trials are recommended to clarify their effects. Our conclusions require confirmation through additional high-quality studies.

## Data Availability

The original contributions presented in the study are included in the article/supplementary material, further inquiries can be directed to the corresponding authors.
